# Aortic Limited Intimal Tear in a 16-Year-Old Boy

**DOI:** 10.5334/jbsr.3547

**Published:** 2024-03-29

**Authors:** Martin Fievez, Ana Falticeanu, Olivier Lebecque

**Affiliations:** 1 Université catholique de Louvain, CHU UCL Namur, Department of Radiology, 1 Avenue Dr G Thérasse, 5530, Yvoir, Belgium; 2 Université catholique de Louvain, CHU UCL Namur, Department of Radiology, 1 Avenue Dr G Thérasse, 5530, Yvoir, Belgium; 3 Université catholique de Louvain, CHU UCL Namur, Department of Radiology, 1 Avenue Dr G Thérasse, 5530, Yvoir, Belgium

**Keywords:** limited intimal tear, acute aortic syndrome, dissection, CTA

## Abstract

*Teaching point:* While demanding urgent management, limited intimal tear (LIT), a rare subtype of acute aortic syndrome (AAS), poses challenges in terms of accurate and timely diagnosis.

## Case History

A 16-year-old male with no prior medical history sought medical attention due to mild spontaneous chest pain. The position and breathing-related pain were suggestive of pericarditis, associated with a mildly increased C-reactive protein (CRP), a discreet ST elevation, and a context of respiratory syncytial virus (RSV) infection. Pain resolved within 48 hours with non-steroidal anti-inflammatory drugs (NSAID). A follow-up transthoracic ultrasound 10 days later revealed a dilation of the aortic root (47 mm) extending to the ascending aorta (AA).

**Figure 1:**
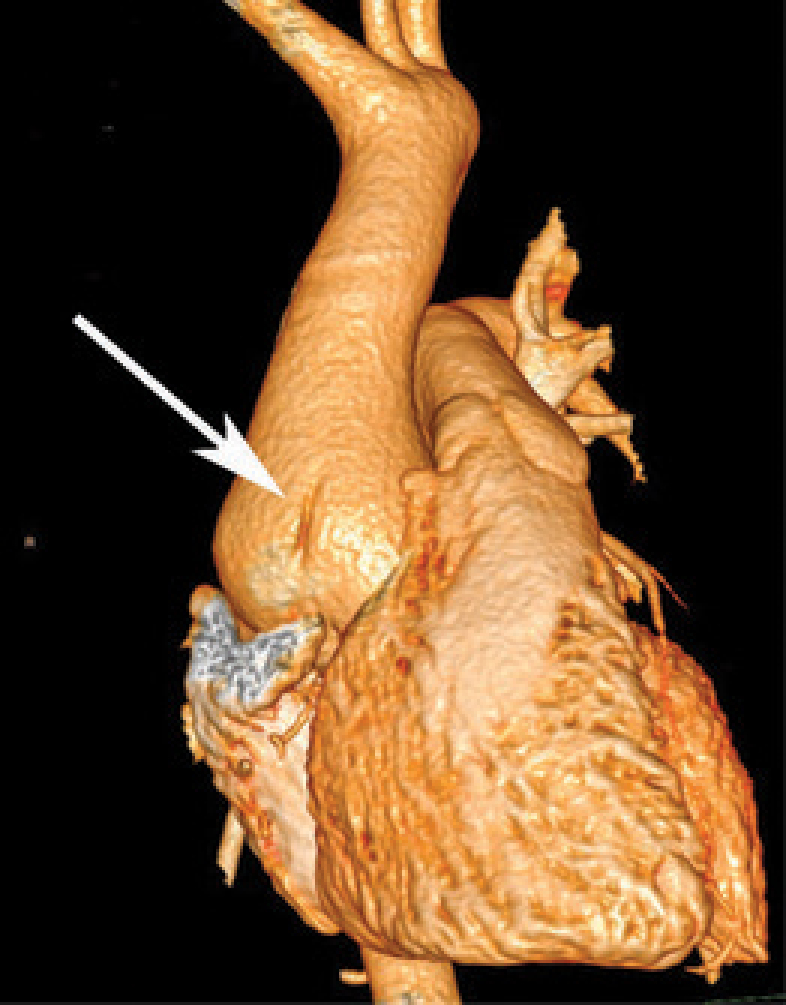
Volume rendering of an aortic CTA showing bulging of the wall.

**Figure 2:**
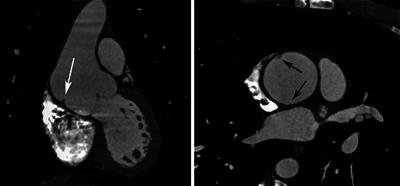
Aortic CTA showing ascending aorta dilatation with an intraluminal parietal band.

Aortic CT angiography (CTA) confirmed a dilatation of 46 mm at the sinotubular junction level and 49 mm in the AA, with effacement of the sinotubular junction. Bulging of the right anterolateral wall of the AA was seen on volume rendering (Figure 1), along with a discreet linear parietal band inside the lumen on CTA images (Figure 2). No intramural hematoma (IMH) was found. The distal AA appeared otherwise normal.

A diagnosis of a limited intimal tear (LIT) was established, and the patient was urgently referred for surgery.

Anatomopathology confirmed the aortic dissection as well as arterial wall remodelling consistent with Marfan syndrome, subsequently confirmed by genetic testing.

## Comment

Acute aortic syndrome (AASs) encompasses 5 classes: classic dissection (CD) (class 1), IMH (class 2), LIT (class 3), penetrating atherosclerotic ulcer (class 4), and iatrogenic/traumatic dissections (class 5). Even though doctors are generally well aware of AAS, LIT remains an exception, despite being first described in 1973 and officially recognized as a variant of AAS by the ‘European Society of Cardiology’ and the ‘American College of Cardiology Foundation/American Heart Association’ since 2001 and 2010, respectively. This may be attributed to the rarity of this condition, accounting for only 4% to 5% of AAS cases [1].

Lacking a false lumen, LIT on CTA can be subtler than CD. The intimal tear can present a linear or stellate morphology, with tears being circumferential or longitudinal. It usually leads to the dilation of the dissected segment due to an eccentric bulging of the arterial wall.

As for any AAS, LIT is divided into type-A if it involves the aortic root (most frequent) and type-B if it only affects the descending aorta. It can be associated with other subtypes, mainly IMH. Extravascular findings such as pericardial/pleural effusion, mediastinal hematoma, and/or hemothorax may be present.

The early outcome of LIT appears to be consistent with that of other AAS, as established so far by the existing limited case studies. The in-hospital mortality rate is approximately 4.2% (compared to CD—5.4% and IMH—4.1%). The prognosis is worse in type-A [1].

Management of LIT follows the approach for AAS, with urgent surgical repair for type-A LIT and medical management for type-B LIT.
